# Efficacy and Safety of Fentanyl Compared With Morphine among Adult Patients with Cancer: A Meta-Analysis

**DOI:** 10.24248/eahrj.v4i1.617

**Published:** 2020-06-26

**Authors:** Astère Manirakiza, Laurent Irakoze, Sébastien Manirakiza, Prudence Bizimana

**Affiliations:** a Kamenge University Hospital; b Karuzi Fiftieth Hospital; c Chongqing Medical University; d University of Burundi

## Abstract

**Background:**

Cancer pain is experienced by numerous patients; thus, the main pain-relieving opioid analgesics, fentanyl and morphine, are of great importance. However, their analgesic efficacy and safety are different among individuals and are still controversial. The aim of this study was to compare the safety and efficacy of fentanyl and morphine among patients with cancer.

**Methods:**

We performed a meta-analysis by searching PubMed and the Cochrane Library up to 01 April 2019. The search terms were fentanyl, morphine, opioids and cancer pain. All randomised controlled trials comparing fentanyl and morphine were included in the analysis.

**Results:**

Overall, the initial search identified 2970 published studies; among them, 9 studies were included in the efficacy analysis and 8 studies were included in the safety analysis. The oral morphine versus oral transmucosal fentanyl subgroup analysis showed a mean difference(MD)=0.47[Confidence interval(CI):0.35-0.58] with an overall effect, Z=8.10, P<.00001. The outcome of the oral morphine versus nasal/transdermal fentanyl subgroup indicated a MD=0.20[CI:0.3-0.37] with an overall effect, Z=2.24 and P=.02.

For the oral morphine versus buccal/sublingual fentanyl subgroup, the analysis revealed a MD=1.80[CI:1.35-2.25] with an overall effect, Z=7.87 and P<.00001.

The oral morphine versus other forms of fentanyl subgroup showed a MD=0.70[95%CI:0.34-1.06] with the test for the overall effect, Z=3.81 and P=.0001.

Constipation, drowsiness, confusion and dry mouth were more common in the morphine group than in the fentanyl group, with a risk ratio=0.60[CI:0.37-0.97]; 0.93[CI:0.69-1.25]; 0.85[CI:0.23-3.13] and 0.54[CI:0.05-6.43], respectively.

**Conclusions:**

Compared with oral morphine, fentanyl is safer and more effective. Moreover, fentanyl presents fewer side effects than morphine, especially constipation, drowsiness, confusion and dry mouth.

## BACKGROUND

Numerous patients experience Cancer pain, especially in the latest stages of the disease. Cancer pain is a critical problem and one of the most distressing symptoms in cancer patients.^1-3^ For the past years, pain has been reported in 59%, 64% and 33% of patients who underwent cancer treatment, patients with advanced diseases and patients after curative treatment, respectively.^4^

The three most common pain rating scales for pain assessment are; the Numerical Rate Scale (NRS), Visual Analogue Scale (VAS), and Verbal Rating Scale (VRS).^5^ These scales are used to estimate the Pain Intensity (PI) and to assess the efficacy of pain treatment.

Many opioids are used for relieving cancer pain.^6^ Opioids are identified as; low pain, moderate to severe pain opioids. For greater efficacy, a combination of opioid therapies are used.^7^ Several studies have been conducted to assess 1, 2 or more opioids compared to placebo or another opioid.^8,9^ Thus, opioids are widely used in the treatment of many types of cancer pain.^10^ However, patients often suffer from constipation, nausea, and vomiting after administration of opioids.^11^ Thus, the safety and efficacy of cancer pain treatment require further exploration.

Several studies have reported that fentanyl is more efficient than morphine in relieving cancer pain. However, for others, it was suggested that fentanyl was equally effective as morphine and was considered to be the opioid of choice.^9,12,13^ So, the safety and efficacy of cancer pain treatment needs further exploration. The objective of this meta-analysis was to compare the safety and efficacy of fentanyl and morphine among cancer patients.

## METHODS

### Inclusion/ Exclusion Criteria

The eligibility criteria were assessed at 3 levels:

1. The common criteria for safety and efficacy were: a) Randomised Controlled Trials (RCT) or Prospective Studies, b) Comparison between at least fentanyl and morphine, and c) Studies published in English;
2. The specific criteria for efficacy were: a) Pain intensity assessed at least 3 times, including baseline; b) Pain rating scales expressed from 0 to 10 points; and c) Studies with outcomes expressed as the means or medians and SD (Standard Deviation) or with a similar inference;
3. The specific criteria for safety were: a) The side effects were a) Pain intensity assessed at least 3 times, including baseline; b) Pain rating scales expressed from 0 to 10 points; and c) Studies with outcomes expressed as the means or medians and SD (Standard Deviation) or with a similar inference;
4. The specific criteria for safety were: a) The side effects were assessed and b) Dichotomous data.

### Search Strategy and Data Extraction

**FIGURE 1.**
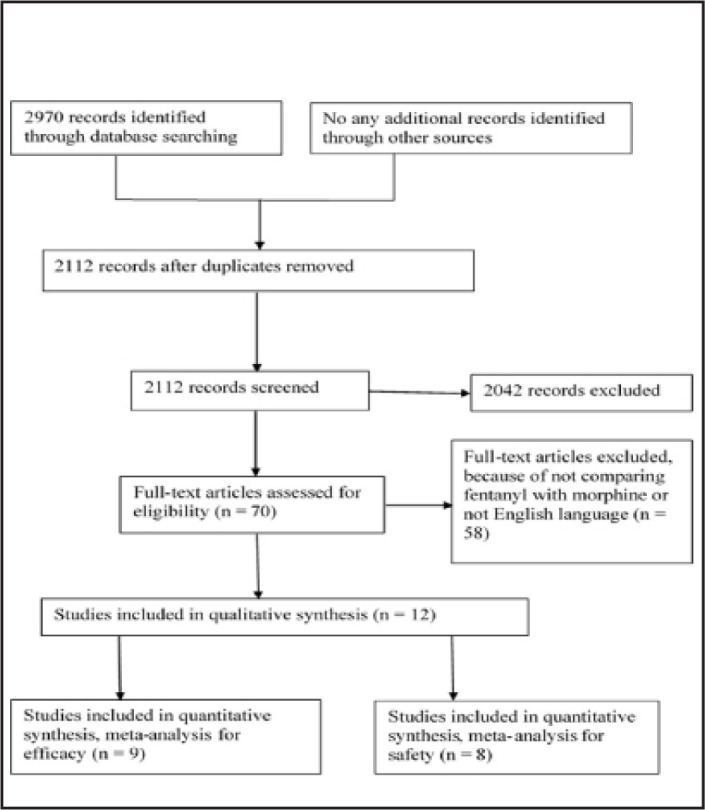
Prisma Flow Diagram

The PubMed and Cochrane Library databases were searched for relevant papers up to 01 April 2019. To identify all relevant studies, we used the search terms ‘‘fentanyl’’ AND ‘‘pain cancer’’ AND ‘‘morphine” OR ‘‘opioids”. A flowchart of the study selection is shown in Figure 1.

### Study Quality and Risk of Bias Assessment

**FIGURE 2.**
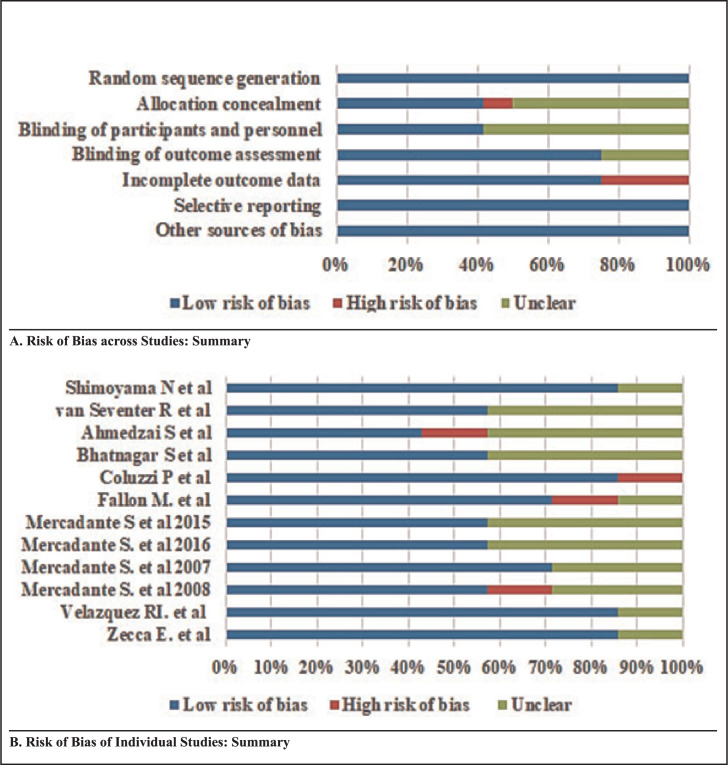
Assessment of Risk of Bias

All of the authors worked independently to search for and assess studies for their methodological quality. The Cochrane Collaboration's tool for assessing the risk of bias was used. This tool included 7 sources of bias: random sequence generation, allocation concealment, blinding of participants and personnel, blinding of outcome assessment, incomplete outcome data, selective reporting and other sources of bias.^14^ The risks of bias across studies are summarised in Figure 2.

### Statistical Analysis

Mean Differences (MD) with 95% Confidence Intervals (CI) were calculated to assess the effect of continuous data, and the Risk Ratio (RR) was calculated for dichotomous data. The MD and RR were pooled using a random effects model to calculate a more conservative result.^14^ Thus, MD>0 indicated a better outcome when using fentanyl, while MD<0 indicated a better outcome when using morphine; RR>1 indicated a high risk of side effects when using fentanyl, while RR<1 indicated a high risk of side effects when using morphine.

For heterogeneity, the estimate of the between-study variance was assessed by I^2^. Therefore, I^2^≤50% might not be significant and I^2^>50% might be significant.^14^

Subgroup analysis was performed according to the mode of drug administration or bioavailability for continuous data and using the type of side effects for dichotomous data. Review Manager (RevMan) [Computer program]. Version 5.3. Copenhagen: The Nordic Cochrane Centre, The Cochrane Collaboration, 2014 was employed for all statistical analyses. Some data across studies was not matched with the statistical study plan. The adjusted data is summarised in Table 2.

**TABLE 1.** Characteristics of Included Studies in the Meta-Analysis

Reference	Year and Country	Study Design	Participants	Time of Assessment	Route of drug administration	
					Morphine	Fentanyl
Bhatnagar S et al. (15)	2014, India	Prospective, Randomised, two arms, open label, active controlled, multi-centric clinical study	186	0,5,15,30,60 minutes	Oral	Oral transmucosal
Coluzzi P et al.(16)	2001, USA	Double-blind, double-dummy, multiple cross-over	84	0,15,30,45,60 minutes	Oral	Oral transmucosal
Fallon M. et al.(17)	2011, Europe-India	Multicenter, randomised, double blinded-double dummy, crossover	79	0,15,30,45,60 minutes	Oral	Pectin nasal spray
Mercadante S. et al.(18)	2015, Italy	Multicenter, randomised, crossover, controlled study	68	0,15.30 minutes	Oral	Buccal tablet
Mercadante S. et al.(19)	2016, Italy	Randomised, crossover, open-label study	45	0,15.30 minutes	Oral	Pectin nasal spray
Mercadante S. et al.(20)	Italy, 2007	Randomised, crossover, controlled study	25	0,15.30 minutes	Intravenous	Oral transmucosal
Mercadante S. et al.(21)	Italy, 2008	Multicenter prospective randomised controlled study	72	0,1,2,3,4 weeks	Oral	Transdermal
Velazquez RI. et al(22)	2014, Spain	Prospective, double-blind, controlled-study	40	0,3,7,15,30 days	Oral	Sublingual tablet
Zecca E. et al(23)	Italy, 2017	Double-blind, double-dummy, parallel-group, non-inferiority RCT	113	0,10,20,30,60 days	Subcutaneous	Sublingual tablet
van Seventer R et al(26)	Netherlands, 2003	Prospective, randomised trial	131	28 days	Oral	Transdermal
NaohitoShimoyama et al(24)	Japan, 2015	Randomised, crossover, double-blinded placebo-controlled and non-blinded active drug controlled, comparative Phase III clinical trial	51	30 and 60 minutes	Oral	Sublingual
Ahmedzai S. et al(25)	UK, 1997	Randomised, open, two-period, crossover study	110	8, 16, 23, and 31days	Oral	Transdermal

Notes: USA: United Nations of America; UK: United Kingdom, RCTs: Randomise Controlled Trials

**TABLE 2.** Pain Rate Scale and Statistical Inference Adjustment

Studies	Scale assessment	Statistical test		Adjustment
Bhatnagar S et al.(15)	NRS/PID	Mean	SD	Subtraction
Coluzzi P et al.(16)	NRS/PID	Mean	SD	SD from Jandhyala et al(9), Subtraction
Fallon M. et al.(17)	NRS/PID	Mean	SEM	Mean and SEM estimated from figure by using Get Data Graph Digitizer software and then, Subtraction
Mercadante S. et al.(18)	NRS/PI	Mean	SD	None
Mercadante S. et al.(19)	NRS/PI	Mean	SD	None
Mercadante S. et al.(20)	NRS/PI	Mean	CI	SD from CI
Mercadante S. et al. 2008 (21)	NRS/PI	Mean	Range	SD from muni-software proposed by Wan X, Wang W, Liu J and Tong T.
Velazquez RI. et al(22)	VAS/PI	Mean	SD	SD estimated from figure by using Get Data Graph Digitizer software
Zecca E. et al(23)	NRS/PI	Mean	SD	None

NRS: Numerical Rate Scale; PID: Pain Intensity Difference; PI: Pain Intensity, SD: Standard Deviation; SEM: Standard Error of Mean, CI: Confident Interval, VAS: Visual Analogue Scale

## RESULTS

### Study Selection and Characteristics

A total of 2,970 records were retrieved from the databases. Of these, 858 were excluded because of duplication. 2,112 studies were screened. Among these, 2,040 were excluded because of inappropriate titles. 70 articles were potentially eligible but 58 of them were removed because they did not compare morphine with fentanyl or were not expressed in English. 5 of the 12 remaining studies had appropriate safety and efficacy assessments. Finally, 9 studies were included in the meta-analysis for efficacy and 8 for the assessment of side effects. The total number of participants in different studies was 1,004 patients.^15-26^ (Figure 1) 1 of the studies was conducted in the USA, 1 in the UK, 1 in Japan, 1 in the Netherlands, 1 in India, 1 in Europe and Indian, 1 in Spain and 5 were conducted in Italy. The study period varied between 1997 to 2017. Some of the study characteristics are shown in Table 1.

### Risk of Bias within Studies

The risk of bias within the included studies (Figure 2. B) showed that most of the studies had a low risk of bias.

### Efficacy Assessment(Figure 3)

**FIGURE 3.**
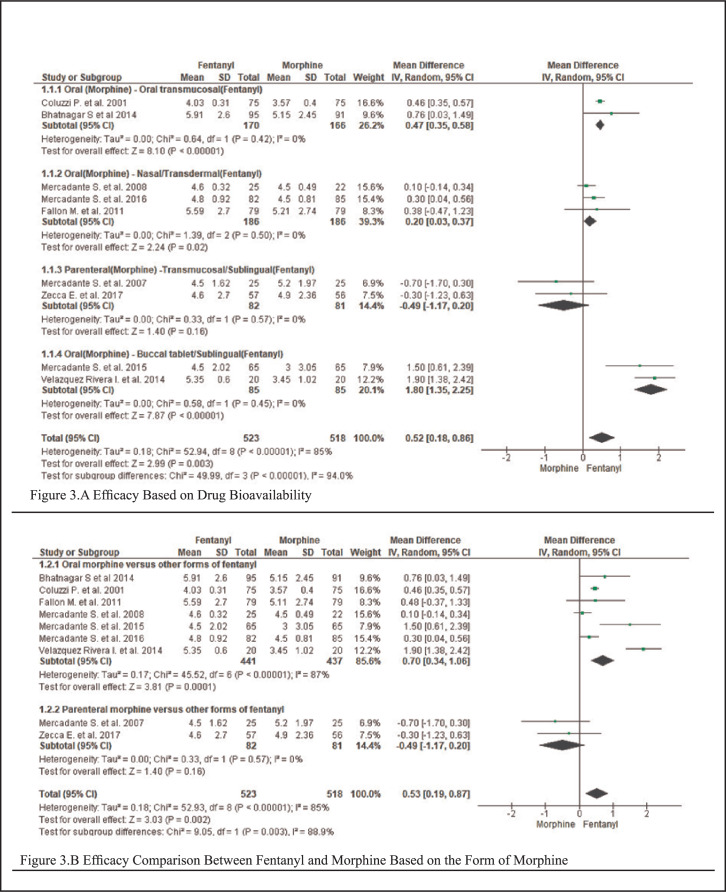
Forest Plots Comparing Efficacy Between Fentanyl and Morphine

When comparing oral morphine to oral transdermal fentanyl, fentanyl had a better outcome than morphine in relieving pain, with a mean Difference (MD)=0.47 [95% Confidence Interval (CI): 0.35-0.58]. The heterogeneity was not significant (I^2^=0% and P=.42). For the test of the overall effect, Z=8.10 and P<.00001.

Considering oral morphine versus nasal and transdermal fentanyl, fentanyl was superior to morphine, MD=0.20 [95%CI:0.03-0.37]. The heterogeneity was not significant (I^2^=0% and P=.50), and for the overall effect, Z=2.24 and P=.02.

Fentanyl (buccal and sublingual) was superior to oral morphine in relieving pain, MD=1.80 [95%CI:1.35-2.25]. The test for the overall effect showed Z=7.87 and P<.00001. The heterogeneity was not significant (I^2^=0% and P=.45).

However, compared with parenteral morphine (intravenous and subcutaneous), fentanyl (transmucosal and sublingual) had lower effectiveness than morphine, MD=0.49, [95%CI:-1.17-0.20]. The heterogeneity was not significant (I^2^=0% and P=.57). For the overall effect, Z=1.40 and P=.16.

Fentanyl is still more efficient than morphine when oral morphine was compared with other forms of fentanyl, MD=0.70, [95%CI:0.34-1.06]. The test for the overall effect showed Z=3.81 and P=.0001. The heterogeneity was significant (I^2^=87% and P<.00001).

### Assessment of Common Side Effects Assessment (Figure 4)

**FIGURE 4.**
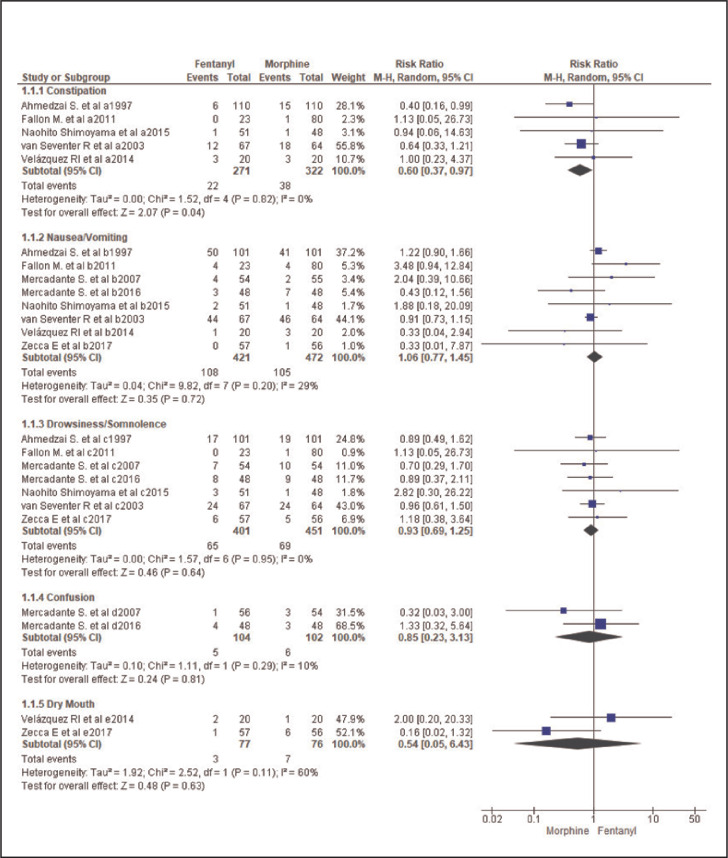
Forest Plot Comparing Fentanyl and Morphine's Side Effects

For common side effects, the assessment showed that constipation appeared more commonly in the morphine group than in the fentanyl group with a Significant Difference, RR=0.60 [95%CI:0.37-0.97], and the heterogeneity was not significant (I^2^=0, P=.82). For the overall effect, Z=2.07 and P=.04.

Drowsiness, confusion and dry mouth seemed to be more common in the morphine group than in the fentanyl group. Their respective RRs was 0.93, [95%CI:0.69-1.25] 0.85, [95%CI:0.23-3.13] and 0.54 [95%CI:0.05-6.43]. However, the difference was not statically significant. There was no significant heterogeneity between studies assessing drowsiness (I^2^=0% and P=.95) and between those assessing confusion (I^2^=10% and P=.29). The tests for the overall effect showed the following effects: drowsiness, Z=0.46 and P=.64; confusion, Z=.24 and P=.81. The heterogeneity between studies assessing dry mouth was moderately significant (I^2^=60%, P=.11). For the overall effect, Z=0.48 and P=.63.

By contrast, nausea/vomiting seemed to be dominant in the fentanyl group, without statistical significance. Indeed, RR=1.06, [95%CI:0.77-1.45]. The heterogeneity was not significant (I^2^=29% and P=.20), and the test for the overall effect showed Z=0.35 and P=.72.

## DISCUSSION

According to this meta-analysis, fentanyl relieved cancer pain better than morphine. The better effectiveness of fentanyl was evident when oral morphine was compared with other forms of fentanyl. When parenteral morphine was compared with other forms of fentanyl, morphine was more effective than fentanyl. However, this efficacy was not statistically significant.

It was also evident that patients taking morphine more frequently developed constipation than those who took fentanyl. Even drowsiness, confusion and dry mouth were more commonly developed in patients who took morphine, although the difference was not statistically significant.

This meta-analysis comparing fentanyl and morphine might provide some evidence and assistance for physicians and patients with the goal of relieving pain. The results of this study indicated that fentanyl administration should produce better results than oral morphine. This study supports the previous studies that suggested that fentanyl was more effective than morphine in relieving cancer pain. It also supports those that reported that fentanyl presented fewer side effects than morphine. However, this study clarifies some cases in which morphine should be more effective than fentanyl and when fentanyl seems to cause more side effects than morphine. This study should be used as a reference for future studies to clarify conditions under which fentanyl or morphine should be used.

The route of fentanyl administration remains an important point in relieving cancer pain. Indeed, before delivering the drug, physicians should determine the best route of fentanyl administration when they must choose between fentanyl and morphine. The nasal mode's advantage is that the venous outflow of the nasal mucosa bypasses the liver and enters systemic circulation, thereby avoiding the hepatic first-pass effect.^13^ It has been reported that nasal fentanyl is similar to intravenous fentanyl in relation to pain control and the incidence of side effects.^27^

The oral transmucosal fentanyl citrate route provides rapid access into systemic circulation with greater bioavailability. The rapid onset of fentanyl is associated with its short duration of effect, making it an attractive option for the treatment of breakthrough cancer pain.^28^ A recent network meta-analysis indicated that transmucosal fentanyl medications achieved a greater level of pain relief in a shorter time frame than oral morphine.^29^

Sublingual fentanyl is provided as a small tablet that is composed of a combination of active drug particles and water-soluble carrier particles coated with a mucoadhesive agent.^30^ In cluding the spray sublingual form, fentanyl is generally well tolerated and is recommended for use for the management of breakthrough pain in opioid-tolerant adult patients with cancer.^28,31,32^

A transdermal fentanyl formulation has been in clinical use since the 1990s.^33^ It is used in palliative care and cancer pain.

A transdermal fentanyl formulation has been in clinical use since the 1990s.^33^ It is used in palliative care and cancer pain. Lower rates of constipation have been demonstrated in terms of side effects, even in patients with terminal cancer.^34^

Subcutaneous delivery of fentanyl has been considered interchangeable with the intravenous route and presents a low incidence of adverse effects.^35^ For patients undergoing caesarean section, a recent study found that subcutaneous fentanyl is an effective alternative to intravenous and intranasal routes of administration for pain management.^27^

Intravenous fentanyl has a duration of analgesia comprise between 30 and 60 minutes after an intravenous bolus.^13^ Intravenous fentanyl can be delivered in a continuous infusion for the treatment of cancer pain in patients requiring high doses for patients who become refractory to other opioids or when other opioids cause intolerable side effects.^36^

Several studies concerning the use of buccal fentanyl in cancer have been conducted. These studies indicated that buccal fentanyl is well tolerated and may improve patient functioning, mood, and overall satisfaction in the management of breakthrough cancer pain.^37-40^

The addition of intrathecal fentanyl to spinal anaesthesia decreases opioid consumption during the period of highest anal-gesic demand after caesarean section.^41^

The transpulmonary fentanyl route remains an experimental phase for the management ofacute or chronic pain. Its duration of action and half-life appear to be prolonged compared to intravenous fentanyl.^28,42^

In our meta-analysis, the included studies had some common points in the constitution of subgroups (Figure 2). Indeed, in the first subgroup (oral morphine versus transmucosal fentanyl), fentanyl had the same properties (transmucosal). In the second subgroup (oral morphine versus nasal and transdermal fentanyl), the nasal and transdermal routes for fentanyl were similar.^13,43^ In the third subgroup (intravenous and subcutaneous morphine versus transmucosal and sublingual fentanyl), morphine was delivered in a route other than oral. For the fourth subgroup (oral morphine versus buccal tablet and sublingual fentanyl), the main common point was oral morphine.

The baseline of PI was not the same when comparing fentanyl with morphine and ranged from 8.40 to 4.8 for fentanyl and from 7.85 to 4.8 for morphine, which also explain the difference regarding pain relief.

There are two main findings of this meta-analysis. First, fentanyl is more effective in relieving pain among patients with cancer than oral morphine. Second, morphine causes more side effects, especially constipation, than fentanyl.

The results of this meta-analysis must be interpreted with caution. In fact, the mean of each study was a difference between two means (the baseline and final assessment means), indicating that the lower the mean difference, the lower the drug efficacy. This difference was the main challenge when interpreting continuous data in the meta-analysis, especially when continuous outcomes were measured in pre- and post-interventions.^14,44^

## CONCLUSION

This meta-analysis outlines the superior efficacy of fentanyl compared with morphine in relieving cancer pain. This meta-analysis clarifies that morphine causes more adverse events, especially constipation, than fentanyl. Fentanyl should be recommended for relieving cancer pain as a first-line drug and the route of drug administration must be considered. However, more studies are still needed for the generalisation of these findings. For example, comparisons between intravenous fentanyl versus intravenous morphine or other combinations should further clarify when to use these two analgesics.

### Limitations

There was only a small number of included studies, and in some studies, all of the necessary data were not provided, as shown in Table 2. The sample size of the 12 included studies was small, and 5 of the included studies were conducted in the same country.
